# The theoretical and empirical basis of a BioPsychoSocial (BPS) risk screener for detection of older people’s health related needs, planning of community programs, and targeted care interventions

**DOI:** 10.1186/s12877-018-0739-x

**Published:** 2018-02-17

**Authors:** Zoe J.-L. Hildon, Chuen Seng Tan, Farah Shiraz, Wai Chong Ng, Xiaodong Deng, Gerald Choon Huat Koh, Kelvin Bryan Tan, Ian Philp, Dick Wiggins, Su Aw, Treena Wu, Hubertus J. M. Vrijhoef

**Affiliations:** 1 Johns Hopkins University, Center for Communication Programs, 111 Market Place, Suite 310, Baltimore, MD 21202 USA; 2 Saw Swee Hock School of Public Health, National University of Singapore, Singapore, Singapore; 3 Department of Global Health and Development, London School of Hygiene and Tropical Medicine, Faculty of Public Health & Policy, London, England; 4 Tsao Foundation, 298 Tiong Bahru Road, #15-01/06 Central Plaza, Singapore, 168730 Singapore; 5 Ministry of Health, College of Medicine, Building 16 College Road, Singapore, 169854 Singapore; 6 University of Stirling, Stirling, UK; 7 UCL Institute of Education, IOE - Social Science, University College London, 20 Bedford Way, London, WC1H 0AL England; 8 Department of Patient & Care, Maastricht University Medical Center, Maastricht, the Netherlands; 9 Vrije Universiteit Brussels, Laarbeeklaan 103, 1090 Brussel, Belgium; 10 Panaxea, Matrix II, unit 1.08/9, Science Park 400, 1098 XH Amsterdam, The Netherlands; 11 Singapore, Singapore

**Keywords:** Interdisciplinary theory, Successful ageing, Risk stratification, Measurement study, Implementation science, Integrated care delivery in the community

## Abstract

**Background:**

This study introduces the conceptual basis and operational measure, of *BioPyschoSocial (BPS) health* and related risk to better understand how well older people are managing and to screen for risk status. The BPS Risk Screener is constructed to detect *vulnerability* at older ages, and seeks to measure dynamic processes that place equal emphasis on Psycho-emotional and Socio-interpersonal risks, as Bio-functional ones. We validate the proposed measure and describe its application to programming.

**Methods:**

We undertook a quantitative cross-sectional, psychometric study with *n* = 1325 older Singaporeans, aged 60 and over. We adapted the EASYCare 2010 and Lubben Social Network Scale questionnaires to help determine the BPS domains using factor analysis from which we derive the BPS Risk Screener items. We then confirm its structure, and test the scoring system. The score is initially validated against self-reported general health then modelled against: number of falls; cognitive impairment; longstanding diseases; and further tested against service utilization (linked administrative data).

**Results:**

Three B, P and S clusters are defined and identified and a BPS *managing score* (‘doing’ well, or ‘some’, ‘many’, and ‘overwhelming problems’) calculated such that the risk of problematic additive BPS effects, what we term health *‘loads’*, are accounted for. Thirty-five items (factor loadings over 0.5) clustered into three distinct B, P, S domains and were found to be independently associated with self-reported health: B: 1.99 (1.64 to 2.41), P: 1.59 (1.28 to 1.98), S: 1.33 (1.10 to 1.60). The fit improved when combined into the managing score 2.33 (1.92 to 2.83, < 0.01). The score was associated with mounting risk for all outcomes.

**Conclusions:**

BPS domain structures, and the novel scoring system capturing dynamic BPS additive effects, which can combine to engender vulnerability, are validated through this analysis. The resulting tool helps render clients’ risk status and related intervention needs transparent. Given its explicit and empirically supported attention to P and S risks, which have the potential to be more malleable than B ones, especially in the older old, this tool is designed to be change sensitive.

**Electronic supplementary material:**

The online version of this article (10.1186/s12877-018-0739-x) contains supplementary material, which is available to authorized users.

## Background

‘There is no single variable that can be used to describe health, and health cannot be measured directly’ [1]. We aim to define - both conceptually and empirically - *BioPsychoSocial (BPS) health* function and related risks in the context of ageing, and to map it to specific health-related outcomes including falls, cognitive impairment, burden of longstanding diseases, and reliance on tertiary care. In so doing, we take two previously tested questionnaires [2–5] and carry out a secondary empirical evaluation to adapt and combine their items into a condensed scoring system for use in capturing BPS health risk and managing status. This work is conceived through dialogue with an interdisciplinary team (programmers, clinicians, government officials, social scientists and psychometricians) for the purposes of improving program implementation. It is based on a dynamic model of health functioning.

Specifically, we seek to better understand risk status of older people by measuring BPS health *loads* that weigh them down, so as to best intervene to help protect or use program *levers* that will buffer, or *lift* people, out of harm’s way as they age. We draw on a case detection pilot intervention in Singapore, which is part of the Community for Successful Ageing (ComSA) [6] initiative. ComSA consists of a two pronged, yet overarching, intervention which offers Community Development courses and activities for proactive Third agers [7] and Care Management for more vulnerable Fourth agers [8]. By Third Agers we refer to those older people who are post-retirement and living the classically defined period between mostly having given up working life and onset of the Forth Age, where limiting health conditions and other adversities conflate to make independent life harder.

ComSA aims to help older people to maintain health for as long as possible, also helping them to build up reserves of resources and support, and to adapt to inevitable changes when they do come. This positioning of *successful ageing*, is outlined in the ComSA Program Framework, Additional file 1: Figure S1. and sits within a longstanding tradition of defining and striving for successful ageing to be a dynamic and evolving process, as elaborated below. Although the program implementation has followed a broad dichotomising of service platforms, targeting largely more proactive health maintenance programming to those already managing well, and adaptive services to those who are more vulnerable, health experiences are not always so neatly divided. Therefore, the ComSA delivery system also allows for some shifting between services and activities across these two platforms, as appropriate.

Our measurement approach, using the BPS Risk Screener, is also embedded within the ComSA program framework. The measure helps to allocate programme participants to services and to meaningfully tailor interventions using an evidenced based delivery system.

To explicate this approach, we present its theoretical and empirical underpinning in three parts. First, we situate this work within the context of literature on healthy and successful ageing. Building on this, we define the B, P, and S domains, and how they can combine to make a person vulnerable at older ages, and position our approach in relation to frailty and related measures. Secondly, we operationalize the three domains in term of questionnaire items, as well as show how we will capture their interrelationships using a dynamic scoring system. We then validate the BPS Risk Screener against health-related outcomes. Finally, we discuss our findings in relation to existing instruments for capturing health and quality of life at older ages.

### Successful ageing

Recent conceptualizations of successful ageing increasingly move away from defining it as simply living longer while staving off disease and biological decline [9–11]. A systematic review of the literature on successful ageing from 1987 to 2013 [11] identified 16 out of 67 articles as arguing for a more holistic definition for successful ageing, and for these to include psychological and social domains. For example, they cite Young et al. [12] definition of successful ageing as the ability to use physical and social adaptive strategies to ‘achieve a sense of well-being, high self-assessed quality of life, and a personal sense of fulfilment, even in the presence of disability and illness’. This idea of adapting and compensating for losses as we age, earlier described by Baltes & Baltes [13], similarly underlies Kahana and Kahana’s proactivity model [14, 15].

The proactivity model emphasizes having and using social, financial and psychological resources, to adapt to stressors in old age and maintain quality of life [14]. These ideas reflect the notion of *active ageing* advanced by the WHO [16, 17]. This is defined as ‘the process of optimizing opportunities for health, participation and security in order to enhance quality of life as people age’, which emphasizes actions across multiple sectors, with the goal of keeping older people in their communities, and contributing to building social capital [17]. Put together, this body of literature echo’s Antonovskey’s longstanding conception of *salutogenesis*, or health as more than simply the absence of disease, but a dynamic process whereby psychosocial asset can promote recovery, or sustain physical health [18]; a view also supported by George Engle in his classic positioning of the Biopsychosocial model [19].

A more critical stance on these concepts also needs to acknowledge the unequal access to resources, and marginalization of some older adults, across differing sociocultural contexts [20–22]. Capturing risks through a comprehensive BPS risk screener (identifying who is vulnerable, as much as how to capture vulnerability) can render not only needs but lived inequalities of older people more transparent for intervention, policy and planning. Our approach draws upon this history of theory and research to ultimately operationalize the dynamic nature of ageing and health. It acknowledges that the notion of a disease-free old age is unrealistic for many individuals [8, 23], and that access to resources and the ‘starting points’ for intervention are unique and will differ for each person.

### Defining BPS health and related risks at older ages

We describe *BPS health* after the World Health Organisations (WHO) [24, 25], as: ‘a set of *dynamic features* [emphasis our own] and dimensions that can be measured’. This definition also accounts explicitly for homeostasis, meaning a system or person’s ability to ‘recalibrate’ or ‘bounce back’, in the face of disruptions. As such, following Huber et al. [25], health is defined as follows:

- *Biological health* (B): ‘In the physical domain, a healthy organism is capable of ‘allostasis’—the maintenance of physiological homoeostasis’.
- *Psychological health* (P): ‘In the mental domain [factors] that contributes to a successful capacity to cope, recover from strong psychological stresses.
- *Social health* (S): ‘In the social domain, people’s capacity to fulfil their potential and obligations’; ‘to participate in social activities’.

Thus, risk is conceived as: the presence of Bio-functional degeneration that can result in either cognitive (dementia), physical (illness) or impairments (limiting activities of daily living); as well as poor Psycho-emotional function and coping resulting in a lack of capacity to recover from strong psychological stresses; and inadequate Socio-interpersonal networks in the form of quantity and quality of relationships, lack of support and empowerment, and capacity to live independently. Risks in each BPS domain are both distinct, as well as inter-related as they can add up, or offset one another within and across the domains. This relationship is further elaborated in relation to the *Load-Levers-Lift process*, and used to operationalize the scoring system of the BPS questionnaire items.

### Vulnerability

*Vulnerability* is explicitly distinguished from *frailty*. Vulnerability, a term transposed from developmental studies [26] can ensue in an older person from any unique BPS combination. It is simply, and broadly conceived as an older person’s risk of being unable to manage. For example, an older person may have limiting long-standing illness but many psycho-social buffers, or strong social support and emotional coping. On the other hand, an older person who is physically robust, may have many P and S problems such as social isolation or depression. According to our definition of vulnerability, an older person who is physically unwell but otherwise robust will not be vulnerable. Conversely, they can be vulnerable without being physically unwell.

Therefore, our concept of vulnerability differs from frailty, which has primarily been associated with bio-functional states, such as slow walking speed, tiredness, weakness and decreased physical activity [27], and has thus been termed a geriatric syndrome [28]. Some frailty instruments have been developed to capture B and some P and S item, and to be inclusive and multifaceted [29, 30] such as the Tilburg Frailty Indicator or TIF [31]. Nevertheless, recent systematic reviews of frailty instruments [32, 33] show frailty tools are still mainly positioned to capture chronological age-related bio-medical deteriorations. For instance, the Physical Frailty Phenotype instrument [34] is the most popular instrument catalogued in a 2016 review [33]. All identified frailty instruments indexed health problems at older ages or measured a latent concept frailty, and none of them sought to capture dynamic effects.

### Loads-levers-lifts processes

The Loads-Levers-Lifts process (Additional file 2: Figure S2) describes a ‘see-saw’ effect in which poor health in the various BPS domains can be conceptualized as loads, making an older person vulnerable. While fewer loads in each BPS domain will protect the person, and provide a better starting point. As loads accumulate across domains, *adaptive capability* of the older person will start to be depleted. We define adaptive capability as having the resources and ability to maintain and overcome health-related deficits. This will help strengthen a person’s basic intrinsic capacity [17] in the face of inevitable changes at older ages. This phenomenon can also be likened to filling a protective basket with a constellation of lifts, as depicted in Additional file 2: Figure S2. The heavier the loads dragging people down to start with, the more the basket needs filling to help people manage successfully into and during the Fourth Age.

Levers are targeted interventions that help to fill these baskets, and keep loads as light as possible, thus building adaptive capability. Our proposed model follows the resilience research tradition [23, 26, 35–39] which clearly positions successful ageing as a dynamic and modifiable process. *Resilience* is defined as ‘doing well despite adversity’ [37–39] and will be achieved when loads are meaningfully offset. Resilience is an important concept in the approach that we propose, with a long history as a research tradition [40]. Resilience in developmental studies can be described as an accidental discovery, that came about when researchers studying children growing up in very vulnerable conditions, were surprised to find a certain proportion of them consistently, and across studies, surpassed all expectations.

This discovery lead to the quest to identify exactly how, and why these children were special. It has since become evident that this ‘ordinary magic’ [35] will be reproduced at any age, across the whole life course, and in the face of varying adversities. Studies of resilience at older ages for example demonstrate how resilient older people can be identified [23, 37–39] and their ageing experiences distinguished from more vulnerable ones [36, 37]. Our research takes the stance that what underscores the resilience phenomenon is not so much an invisible constellation of protective traits, but being able to leverage enough lift to tip back the balance in an individuals’ favour. The starting point to rendering these processes explicit will be to accurately capture individual loads, and hence this construct, is the focus of the current study.

### Measuring ‘loads’: A theoretically derived scoring system

The BPS Risk Screener ranges capturing scores on those who are managing or ‘doing well’, to those that are at risk or living with ‘overwhelming problem’, in other words detecting the range or ‘weight’ of health loads. This range is captured in a unique scoring system that forthcoming analyses validates. The scoring approach was constructed iteratively from our theory base, pilot administration in the community, and statistical scoping and sensitivity analysis to find the best way to simply yet meaningful capture the BPS interrelations. Additional file 3: Figure S3a-b provides a schematic to explain the scoring structure.

We derive ‘some’ vs. ‘a lot’ of health problems benchmarks from population norms (statistical averages) within the individual B, P, and S domains to determine what we call *managing counts,* see 3a. We then sum managing counts across individual domains into an overall managing *score*, see 3b, to capture the BPS risk categories. The term ‘managing’ is chosen to report the scores because it was judged more sensitive than the term ‘risk’ score when sharing results with older people themselves.

### Specific objectives

The objectives of the forthcoming empirical validation of the Risk Screener are threefold:

1. To identify the specific BPS factors and related items that map to our theoretically defined domains;
2. To examine individual B, P, and S predictors and additive BPS effects in relation to our theoretical stance that health is multi-faceted and self-reported health would therefore be significantly associated with all three domains;
3. To further examine associations of our managing/risk scores with: falls, cognitive impairment, burden of longstanding diseases, and reliance on tertiary care outcomes.

## Methods

### Measuring additive effects

This is a psychometric study, using a quantitative survey design to examine the theoretical premise of Risk Loads as an operational construct. Risk Load measurement must account for ‘dosing’ of loads that add up to make things worse, both within and across BPS domains. The scoring system is based on our theoretical starting point. First, we calculate BPS domains managing counts: 0 ‘none’, 1 ‘some’, and 2 ‘a lot’. For the B and P domains ‘None’ is scored when no risk items are ticked; thresholds of ‘some’ and ‘a lot’ were derived at or above the median. Except for the S domain, which was divided into tertiles. Next, we sum across BPS domains: adding the managing counts to reflect the following: 0–1 ‘doing well’; 2–3 ‘some problems’; 4–5 ‘many problems; 6 ‘overwhelming problems’.

### Data collection and setting

Data were collected during August to October 2014, using non-randomized convenience sampling, and listings of addresses of participants of 60 years of age and older residing in Whampoa, a neighbourhood in Singapore with a high density of older people (*N* = 1375). The questionnaire was translated and administered in English, Mandarin, and Malay. Surveyors were familiar with Chinese dialects such as Hokkien and Cantonese and using scripts adapted the questions orally when necessary. The survey was carried out using door-to-door knocking, mainly in public Housing Development Board (HDB) flats. Public housing policy in Singapore equitably distributes these flats amongst ethnicities (predominantly Chinese, with fewer Malays, Indians); more than 90% are owner occupied although a minority who are socio-economically deprived may rent them from HDB.

These analyses are derived from a secondary data source. The data were originally collected by Tsao Foundation funded by the Ministry of Health of Singapore using a contracted survey company experienced in collecting data from older people in community settings, and trained in inter-RAI (the decision-making observed test for cognitive impairment) [41]. Older people were approached directly, or though family members / care givers, who were first involved in the recruitment process where judged necessary. The data collectors followed a standard operating procedure, involving first gaining agreement from the care giver to invite the more notably vulnerable to take part in the survey, including testing for cognitive impairment with inter-RAI,

All participants themselves were also explained the purpose of the data collection, invited to take part, and asked for their own signed consent. Regardless of survey participation, further information and follow-up on care services by Tsao Foundation was made available. Outdoor community events such as ComSA ‘tea parties’ outlining Tsao Foundation Community Development services were made open to care givers and older people alike. A hospital referral scheme for Care Management was also established.

### Ethical review

The National University of Singapore (NUS) study team was granted an exemption certificate by NUS’ Institutional Review Board (IRB) to use the community survey as a secondary data source. An amendment was approved to analyse an expanded de-identified dataset linked by Ministry of Health, containing admissions data.

### Measures and outcomes

The survey was designed to collect socio-demographic information, basic health and functional data, and thus contained the EASYCare standard 2010. EASYCare is positioned as a global tool [42] with a long history as a leading, comprehensive, geriatric questionnaire useful in helping to assess clinical needs and to open discussion for social or practical support, one-on-one with clients. It can be valuably used for programmers to assess each individual and then agree with them what they would like in terms of support, referrals or other types of help. Equally, it was originally designed to derive summary scores of health which could be used to compare health status regionally, nationally, globally. The current analysis builds on this latter approach, using a specific definition of health and risk.

The EASYCare items were themselves derived from the array of geriatric assessment tools [43], and account particularly well for limitations in ADLs. This tool inclusively asks about P, feelings and depression. Potential S indicators focused on loss of B independence, such that might come with decline in cognitive ability, for example not being able to manage ones’ affairs, or having carer support. Other intended S items, described fully later, transpired empirically as emphasizing the psychological. For instance, asking if clients ‘felt’ there were people they could call on for help. Responses to EASYCare items were either binary or Likert.

Since detail on quality and quantity of social networks are not reflected in the EASYCare item list, the Lubben Social Network Scale, which measures frequency of contact and quality of relationships with friends, and family (excluding those that you live with) were added to the questionnaire; these data are collected as counts, i.e. the number of family, and friends, in contact ‘at least once a month’.

In addition, the survey contained the following self-reported outcomes: health status (poor to excellent); number of chronic or longstanding diseases (including: heart disease, stroke, chest/lung disease, cancer, arthritis, osteoporosis and bone fractures, diabetes, high blood pressure, high cholesterol, and doctor diagnosed depression); falls in the last year (none, one, and two or more); number of hospital admissions rates in the last 6 months. It also contained: an observable Inter-RAI cognitive function test (the decision-making observed test, ranging from intact, mild, moderate to severe impairment) [41]. Administrative data on hospital admissions, presenting at Emergency Department (ED), and Length of Stay (LoS), all in the last 6 months were added via data linkage.

### Data analysis

Analysis sought to examine the comprehensive BPS items list, to empirically identify items within each cluster, to then adapt them and test the structure of our theoretically derived scoring schema, for use in stratified care planning. Analyses were performed in R, a software environment for statically computing and graphics. We initially screened the data by examining inter-item correlations using Pearson’s product moment correlation coefficient, the Kaiser-Meyer-Olkin (KMO) measure of sampling adequacy and Bartlett’s test of sphericity to determine its suitability for factor analysis [44]. A sensitivity analysis was also used, fitting the test to a polychoric correlation [45–47]. We confirmed a KMO value greater than 0.50 and a significant Bartlett’s test to proceed with the factor analysis [48, 49].

Exploratory Factor Analysis was then applied to reduce the number of BPS indicators from an initial 52 EASYCare and Lubben items. As these 52 items were ordinal variables, we used polychoric measure [48, 50] to compute the correlation between the items, using Promax Rotation specifying three components. We iteratively removed items with the weakest contribution to the factor structure at each step until all items had a primary factor loading of 0.5 or above across all components, and all cross-loadings were 0.3 and below. The Horn’s parallel analysis [51–53] and the cumulative variance is reported. We examined internal consistency using ordinal alpha [54].

We tested the scoring system, looking for evidence of dose response across BPS domains by first examining gradients associated with our managing categories against outcomes of interest. Multivariate logistic regression was used on the outcomes that were dichotomised, and Poisson for outcomes of count data, first examining associations with the individual BPS managing counts, then the BPS managing score itself. The linear trend for each domain is examined by treating the BPS domains managing counts as numerical variables by coding ‘none’, ‘some’, and ‘a lot’ as 0, 1 and 2. For the managing score we coded ‘doing well’, ‘some problems’, ‘many problems’, and ‘overwhelming problems’ as 0, 1, 2 and 3 respectively.

Three outcomes were binary: ‘poorer’ self-reported health, corresponds to poor or fair to, and good, very good, or excellent corresponds to ‘better health’; more than one fall in the last 12 months corresponds to ‘yes’, and none corresponds to ‘no’; observed intact cognitive impairment corresponds to ‘not impaired’, and borderline and above to ‘impaired’.

Five outcomes were based on counts data. These were, total number: of self-reported hospitalization and diseases; hospitalization, ED visits, LoS (all administrative data). For the number of self-reported hospitalization and diseases the goodness-of-fit tests suggested adequate fit and hence Poisson modeling is reasonable. For the total number of hospitalization, ED visit and LoS from administrative data, the goodness-of-fit test suggested inadequate fit due to over-dispersion and hence negative-binomial was used.

The managing count individual B, P, S categories were put into the same model which was adjusted for sociodemographic factors. The combined managing score categories were entered in the next model, also adjusted for sociodemographic covariates. These were: sex, female vs. male (reference); education, no formal education vs. primary education and above (reference); income ≥ than $250 a month as ‘poor’ vs. above this threshold as ‘not poor’ (reference); age, > 75 vs. ≤75 years (reference); and ethnicity, ethnic minorities vs. Chinese (reference).

## Results

### Sample characteristics

Complete data were available for *N* = 1325 cases on our outcomes. Survey respondents’ socio-demographic characteristics were compared to 2015 census data [55] (Additional file 4: Table S1). In terms of gender, 59% of the Whampoa survey respondents were women compared to 53% in the general population; and 83% of both populations were Chinese, and represented ethnic minorities. Indians were somewhat over represented compared to the general population groups (11% vs. 6%), and somewhat less for Malays (5% vs. 9%). The survey respondents tended to be older (36% over 75 years vs. 25%), and less educated to higher levels (only 9% post-secondary education vs. 18%) compared to the general population. Therefore, our sample was somewhat more representative of those likely to have greater risk profiles, in terms of age and education but broadly matched ethnic and gender profile.

### Clustering of BPS domains

Initially, we examined the factorability of the 52 EASYCare and Lubben items. The KMO measure was 0.93 (Pearson) and 0.58 (Polychoric) and Bartlett’s tests were statistically significant (*p* < 0.05) in both cases. We reduced the 52 potential items to 35 through the iterative factor analysis process, identifying those with the highest predictive value within their clusters, Table 1a-c. Horn’s parallel analysis produced a screen plot with three discernible components. The ordinal alphas suggested strong internal consistency: B (21 items) 0.98; P (8 items), 0.85; S (6 items) 0.80. No substantial improvements in alpha for any of the scales could have been reached by eliminating more items. The cumulative variance was 66%.

**Table 1:** a-c: Factor analyses showing included items, *n* = 1325 study participants over 60 years of age

Questionnaire item list	Component labels and item loadings by domain
1a. Biological	
Can you see (with glasses)…	0.505
Can you hear (with hearing aid)…	0.568
Difficulty in making yourself understood…	0.815
Use the telephone…	0.886
Keep up personal appearance…	0.962
Dress yourself…	0.958
Wash your hands and face…	0.959
Use the bath or shower…	0.988
Do your housework…	0.880
Prepare your own meals…	0.863
Feed yourself…	0.964
Take your own medicine…	0.877
Any accidents with your bladder…	0.712
Accidents with your bowels…	0.736
Use the toilet…	0.973
Move from bed to chair…	0.907
Get around indoors…	0.866
Manage stairs…	0.846
Walk outside…	0.918
Go shopping…	0.884
Any difficulty getting to public services?	0.881
1b. Psychological	
Safe inside your home?	0.573
Feel threatened…	0.532
Feel discriminated…	0.780
Anyone to help in case of illness…	0.611
Happy with accommodation?	0.822
Feel lonely?	0.599
Feeling down…	0.705
Bothered by having little interest or pleasure?	0.727
1c. Social	
How many relatives do you see or hear from at least once a month?	0.750
How many relatives do you feel at ease with that you can talk about private matters?	0.833
How many relatives do you feel at close to such that you could call on them for help?	0.843
How many of your friends do you see or hear from at least once a month?	0.535
How many of your friends do you feel at ease with that you can talk about private matters?	0.654
How many of your friends do you feel at close to such that you could call on them for help?	0.598

The B domain had some of the strongest inter-correlations, which appears to reflect that these were also best developed/worded for our setting, and the most abundant component of the EASYCare tool. Interestingly, we had considered capturing up to two clusters in this domain, a cognitive and a physical/ADLs one. Instead potentially cognitive items such as having ‘difficulty in making yourself understood…’ or ability to do complicated tasks such as prepare meals, take medicine, or neglect personal grooming all clustered in the one domain, alongside more obviously physical ones.

In the S domain, all Lubben items clustered meaningfully together; items reflected both quality and quantity of relations. As for the P domain, emotional items such as feeling down, or those suggestive of effects from abusive relationships such as being discriminated against or not feeling safe, clustered. A P item on not having someone ‘to ask for help’ likely captured helplessness or lack of self-efficacy - feeling unable to ask.

Distributions of the managing score based on the 35 item clusters are shown in Additional file 5: Figure S4. It was positively skewed with 38.7% ‘doing well’, 44.0% had ‘some’, 15.7% ‘many’ and only 1.6% ‘overwhelming’ problems. This last category contained *n* = 21 cases, which limited upcoming analysis at this level.

### Dose response relationships

Additional file 6: Figure S5a-d, Additional file 7: Figure S5e–h, shows gradients and significant *p*-values for trend for at < 0.001 for all outcomes based on an unadjusted analysis. These analyses were sometimes based on low proportions and small averages, e.g. only 11.5% had been admitted to hospital, and just 0.7% more than once; 14.9% of the population reported a having a longstanding disease. Nevertheless, we found that 66.7% of those with overwhelming problems described their own general health as poor, compared to just over a third 35.6% in the many problems category.

Correspondingly, 42.9% of those with overwhelming problems, had self-reported being hospitalized in the last 6 months, sometimes more than once, compared of 17.3% of those with many problems who were only admitted once. The proportion reporting falls showed the smoothest gradient. Nearly half, or 49.0% of those with many problems had moderate or worse cognitive impairment, and 57.1% of those with overwhelming problems did too. We saw a flatter relationship on administratively collected service usage data, which we unpack further in adjusted models; the administrative data although objectively collected may not reflect the range of services captured by the self-report.

Multivariate explanatory analyses are detailed throughout Tables 2, 3, 4, 5, 6, 7, 8,9 and summarized in Additional file 8: Figure S6. Supporting our BPS ‘health’ definition, all BPS domains where found to independently contribute to general self-reported health: B domain Odds Ratio (OR) 1.99 (1.64 to 2.41), P OR 1.59 (1.28 to 1.98), and S OR 1.33 (1.10 to 1.60), and the fit improved when used as a managing score, OR of 2.33 (1.92 to 2.83, < 0.01). It was also consistent with our chosen outcomes that the B domain linear trend was significant for all of them.

**Table 2:** Adjusted regression analysis (logistic, Odds Ratios) for Self-Reported Health, *n* = 1325 participants aged 60 and over

Model	Domain	Level	OR (95% CI, *P*-value)	*P*-value	Linear trend (95%CI, *P*-value)
Model with individual domain managing counts^a^	Biological	No risk	Reference	< 0.01	*1.99 (1.64 to 2.41, < 0.01)*
		Some risk	1.46 (0.99 to 2.15, 0.06)		
		High risk	3.92 (2.68 to 5.72, < 0.01)		
	Psychological	No risk	Reference	< 0.01	*1.59 (1.28 to 1.98, < 0.01)*
		Some risk	1.62 (1.09 to 2.41, 0.02)		
		High risk	2.50 (1.56 to 4.02, < 0.01)		
	Social	No risk	Reference	< 0.01	*1.33 (1.10 to 1.60, < 0.01)*
		Some risk	1.69 (1.17 to 2.46, 0.01)		
		High risk	1.82 (1.24 to 2.67, < 0.01)		
Model with managing scores^b^	BioPsychoSocial	Doing well	Reference	< 0.01	*2.33 (1.92 to 2.83, < 0.01)*
		Some problems	2.18 (1.52 to 3.13, < 0.01)		
		Many problems	4.98 (3.26 to 7.59, < 0.01)		
		Overwhelming problems	17.96 (6.86 to 47.05, < 0.01)		

^a^The individual B, P, S categories were put into the same model with the following covariates: age, gender, ethnicity, income and education

^b^Likewise, the combined managing score categories were put into a model with the following covariates: age, gender, ethnicity, income and education

**Table 3:** Adjusted regression analysis (logistic, Odds Ratios) for Falls in the last 12 months, *n* = 1325 participants aged 60 and over

Model	Domain	Level	OR (95% CI, *P*-value)	*P*-value	Linear trend (95%CI, *P*-value)
Model with individual domain managing counts^a^	Biological	No risk	Reference	< 0.01	*1.66 (1.35 to 2.04, < 0.01)*
		Some risk	1.34 (0.88 to 2.03, 0.17)		
		High risk	2.74 (1.83 to 4.1, < 0.01)		
	Psychological	No risk	Reference	0.043	*1.35 (1.06 to 1.72, 0.01)*
		Some risk	1.53 (1.00 to 2.34, 0.05)		
		High risk	1.70 (1.00 to 2.88, 0.05)		
	Social	No risk	Reference	0.271	*1.18* *(0.97 to 1.44, 0.11)*
		Some risk	1.16 (0.78 to 1.71, 0.46)		
		High risk	1.39 (0.93 to 2.06, 0.11)		
Model with managing scores^b^	BioPsychoSocial	Doing well	Reference	< 0.01	*1.86 (1.52 to 2.28, < 0.01)*
		Some problems	1.96 (1.33 to 2.89, < 0.01)		
		Many problems	3.67 (2.33 to 5.76, < 0.01)		
		Overwhelming problems	5.37 (2.01 to 14.33, < 0.01)		

^a^The individual B, P, S categories were put into the same model with the following covariates: age, gender, ethnicity, income and education

^b^Likewise, the combined managing score categories were put into a model with the following covariates: age, gender, ethnicity, income and education

**Table 4:** Adjusted regression analysis (logistic, Odds Ratios) for Observed Cognitive Function, *n* = 1325 participants aged 60 and over

Model	Domain	Level	OR (95% CI, *P*-value)	*P*-value	Linear trend (95%CI, *P*-value)
Model with individual domain managing counts^a^	Biological	No risk	Reference	< 0.01	*2.86 (2.34 to 3.49, < 0.01)*
		Some risk	1.87 (1.24 to 2.81, < 0.01)		
		High risk	7.66 (5.19 to 11.29, < 0.01)		
	Psychological	No risk	Reference	0.12	*1.26 (1.00 to 1.59, 0.05)*
		Some risk	1.43 (0.95 to 2.14, 0.09)		
		High risk	1.47 (0.88 to 2.44, 0.14)		
	Social	No risk	Reference	< 0.01	*1.51 (1.25 to 1.83, < 0.01)*
		Some risk	1.82 (1.24 to 2.66, < 0.01)		
		High risk	2.35 (1.59 to 3.46, < 0.01)		
Model with managing scores^b^	BioPsychoSocial	Doing well	Reference	< 0.01	*2.78 (2.29 to 3.39, < 0.01)*
		Some problems	2.82 (1.93 to 4.13, < 0.01)		
		Many problems	8.64 (5.62 to 13.3, < 0.01)		
		Overwhelming problems	12.77 (4.95 to 32.91, < 0.01)		

^a^The individual B, P, S categories were put into the same model with the following covariates: age, gender, ethnicity, income and education

^b^Likewise, the combined managing score categories were put into a model with the following covariates: age, gender, ethnicity, income and education

**Table 5:** Adjusted regression analysis (Poisson, Incidence Rates Ratios) for Self-Reported Hospitalization, *n* = 1325 participants aged 60 and over.

Model	Domain	Level	IRR (95% CI, *P*-value)	*P*-value	Linear trend (95%CI, *P*-value)
Model with individual domain managing counts^a^	Biological	No risk	Reference	< 0.01	*1.88 (1.52 to 2.32, < 0.01)*
		Some risk	1.59 (1.03 to 2.47, 0.04)		
		High risk	3.45 (2.27 to 5.23, < 0.01)		
	Psychological	No risk	Reference	0.351	*1.17 (0.93 to 1.48, 0.18)*
		Some risk	1.3 (0.87 to 1.96, 0.20)		
		High risk	1.29 (0.77 to 2.16, 0.33)		
	Social	No risk	Reference	0.50	*0.99 (0.81 to 1.2, 0.89)*
		Some risk	0.81 (0.55 to 1.19, 0.28)		
		High risk	0.97 (0.66 to 1.42, 0.88)		
Model with managing scores^b^		Doing well	Reference		*1.67 (1.37 to 2.02, < 0.01)*
		Some problems	1.49 (1.01 to 2.2, 0.05)		
	BioPsychoSocial	Many problems	2.47 (1.58 to 3.85, < 0.01)	< 0.01	
		Overwhelming problems	5.67 (2.8 to 11.48, < 0.01)		

^a^The individual B, P, S categories were put into the same model with the following covariates: age, gender, ethnicity, income and education

^b^Likewise, the combined managing score categories were put into a model with the following covariates: age, gender, ethnicity, income and education

**Table 6:** Adjusted regression analysis (Poisson, Incidence Rates Ratios) for Number of Diseases, *n* = 1325 participants aged 60 and over

Model	Domain	Level	IRR (95% CI, *P*-value)	*P*-value	Linear trend (95%CI, *P*-value)
Model with individual domain managing counts^a^	Biological	No risk	Reference	< 0.01	*6.02 (4.83 to 7.49, < 0.01)*
		Some risk	2.54 (1.47 to 4.4, < 0.01)		
		High risk	22.59 (14.22 to 35.87, < 0.01)		
	Psychological	No risk	Reference	< 0.01	*1.2 (1.06 to 1.36, < 0.01)*
		Some risk	1.52 (1.22 to 1.89, < 0.01)		
		High risk	1.28 (0.97 to 1.7, 0.08)		
	Social	No risk	Reference	< 0.01	*1.06 (0.94 to 1.19, 0.31)*
		Some risk	1.5 (1.18 to 1.91, < 0.01)		
		High risk	1.19 (0.93 to 1.54, 0.17)		
Model with managing scores^b^	BioPsychoSocial	Doing well	Reference	< 0.01	*2.44 (2.17 to 2.74, < 0.01)*
		Some problems	8.82 (5.44 to 14.29, < 0.01)		
		Many problems	17.3 (10.61 to 28.19, < 0.01)		
		Overwhelming problems	24.63 (13.41 to 45.25, < 0.01)		

^a^The individual B, P, S categories were put into the same model with the following covariates: age, gender, ethnicity, income and education

^b^Likewise, the combined managing score categories were put into a model with the following covariates: age, gender, ethnicity, income and education

**Table 7:** Adjusted regression analysis (Poisson, Incidence Rates Ratios) for Hospitalisation (administrative data), *n* = 1325 participants aged 60 and over.

Model	Domain	Level	IRR (95% CI, *P*-value)	*P*-value	Linear trend (95%CI, *P*-value)
Model with individual domain managing counts^a^	Biological	No risk	Reference	< 0.001	*1.66 (1.4 to 1.96, < 0.01)*
		Some risk	1.72 (1.25 to 2.37, 0.001)		
		High risk	2.75 (1.97 to 3.85, < 0.001)		
	Psychological	No risk	Reference	0.701	*0.92(0.74 to 1.15, 0.477)*
		Some risk	0.99 (0.68 to 1.43, 0.961)		
		High risk	0.81 (0.49 to 1.32, 0.4)		
	Social	No risk	Reference	0.136	*1.13 (0.96 to 1.32, 0.142)*
		Some risk	1.36 (1 to 1.85, 0.053)		
		High risk	1.28 (0.92 to 1.77, 0.144)		
Model with managing scores^b^	BioPsychoSocial	Doing well	Reference	< 0.001	*1.41 (1.19 to 1.67, < 0.01)*
		Some problems	1.39 (1.03 to 1.86, 0.032)		
		Many problems	2.16 (1.49 to 3.13, < 0.001)		
		Overwhelming problems	1.88 (0.74 to 4.78, 0.184)		

^a^The individual B, P, S categories were put into the same model with the following covariates: age, gender, ethnicity, income and education

^b^Likewise, the combined managing score categories were put into a model with the following covariates: age, gender, ethnicity, income and education

**Table 8:** Adjusted regression analysis (Poisson, Incidence Rates Ratios) for presenting at Emergency Department (administrative data), *n* = 1325 participants aged 60 and over.

Model	Domain	Level*	IRR (95% CI, *P*-value)	*P*-value	Linear trend (95%CI, *P*-value)
Model with individual domain managing counts^a^	Biological	No risk	Reference	< 0.001	*1.89 (1.55 to 2.3, < 0.001)*
		Some risk	1.89 (1.29 to 2.75, 0.001)		
		High risk	3.58 (2.41 to 5.3, < 0.001)		
	Psychological	No risk	Reference	0.795	*1.09 (0.85 to 1.39, 0.5)*
		Some risk	1.08 (0.7 to 1.66, 0.743)		
		High risk	1.2 (0.69 to 2.06, 0.522)		
	Social	No risk	Reference	0.001	*1.43 (1.19 to 1.74, < 0.01)*
		Some risk	1.7 (1.17 to 2.47, 0.005)		
		High risk	2.08 (1.41 to 3.06, < 0.001)		
Model with managing scores^b^	BioPsychoSocial	Doing well	Reference	< 0.001	*1.98 (1.61 to 2.42, < 0.001)*
		Some problems	2.3 (1.61 to 3.29, < 0.001)		
		Many problems	4.22 (2.7 to 6.61, < 0.001)		
		Overwhelming problems	4.08 (1.37 to 12.17, 0.012)		

^a^The individual B, P, S categories were put into the same model with the following covariates: age, gender, ethnicity, income and education

^b^Likewise, the combined managing score categories were put into a model with the following covariates: age, gender, ethnicity, income and education

**Table 9:** Adjusted regression analysis (Poisson Incidence Rates Ratios) for Length of Stay (administrative data), *n* = 1325 participants aged 60 and over

Model	Domain	Level	IRR (95% CI, *P*-value)	P-value	Linear trend *(95%CI, P-value)*
	Biological	No risk	Reference	< 0.001	*2.95 (2.17 to 4.03, < 0.001)*
		Some risk	2.39 (1.35 to 4.22, 0.003)		
		High risk	9.01 (4.82 to 16.82, < 0.001)		
Model with individual domain managing counts^a^	Psychological	No risk	Reference	0.171	*1.02 (0.68 to 1.53, 0.926)*
		Some risk	1.63 (0.82 to 3.24, 0.166)		
		High risk	0.6 (0.24 to 1.52, 0.285)		
		No risk	Reference		
		Some risk	1.27 (0.72 to 2.22, 0.409)	0.138	*1.36 (1.01 to 1.83, 0.041)*
	Social	High risk	1.87 (1.03 to 3.37, 0.038)		
Model with managing scores^b^	BioPsychoSocial	Doing well	Reference	< 0.001	*2.93 (2.11 to 4.08, < 0.001)*
		Some problems	4.02 (2.33 to 6.92, < 0.001)		
		Many problems	8.4 (4.04 to 17.48, < 0.001)		
		Overwhelming problems	7.14 (1.07 to 47.66, 0.042)		

^a^The individual B, P, S categories were put into the same model with the following covariates: age, gender, ethnicity, income and education

^b^Likewise, the combined managing score categories were put into a model with the following covariates: age, gender, ethnicity, income and education

However, the domain P did not appear to independently contribute to cognitive function outcomes, although this statistic was borderline OR 1.26 (1.00 to 1.59), nor to hospital admissions. While S was itself also not independently associated with hospitalization, or with falls - perhaps because our social domain does not capture social support inside the home - and number of diseases. Number of diseases captured the largest association in the overall B domain, Incidence Risk Ratio (IRR) of 6.02 (4.83 to 7.49), and some impact from the P domain IRR of 1.2 (1.06 to 1.36, < 0.01).

Unsurprisingly, the decision to admit to hospital appeared driven only by Bio-functional factors: on self-reported outcomes the B domain IRR was 1.88 (1.52 to 2.32), on administratively recorded admissions it was 1.66 (1.4 to 1.96). However, ED presenting and LoS were notably also associated with the S domain: ED S linear trend IRR of 1.43 (1.19 to 1.74), and mounting effect of 1.7 (1.17 to 2.47) up to 2.08 (1.41 to 3.06); and a LoS S linear trend effect of 1.36 (1.01 to 1.83). Moreover, despite the individual BPS domains not always being significant, combined managing scores meaningfully improved associations in the expected direction, at each level, for most outcomes; excepting for service usage administrative data.

For these, we found a flattening of dose response, suggesting that these outcomes were associated with effects mainly between the lower and higher bands (a managing score over 4pts), and that the granular effects of many or overwhelming problems were harder to capture – i.e. not present in administratively collected service usage outcomes. Indeed, for administratively collected hospitalization, ED visits and LoS, those with overwhelming problems did not show poorest performance. This could be explained by administratively collected outcomes not accounting for intermediate or long-term care, or palliative home care, which is likely where the ‘overwhelming’ problems respondents would have sought the help they self-reported.

The self-reported hospitalization outcome itself did capture differences between those with ‘many problems’ IRR 2.47 (1.58 to 3.85) and ‘overwhelming problems’ IRR 5.67 (2.8 to 11.48), and although subject to recall bias may still be more reflective of a range of tertiary service usages.

In sum, our findings are consistent with dose response effects. The linear trend of the managing score was significant, with some fairly sizable effects: number of diseases IRR of 2.44 (2.17 to 2.74); observed cognitive function OR of 2.7 (2.29 to 3.39), and significant effects on self-reported health. These higher magnitude associations support our theoretical stance and conceptual starting-point of the tool as measuring the latent concept of BPS health.

## Discussion

This work is distinguishable from other health, and frailty measures for older people [32, 33, 56] by its interdisciplinary and empirically tested classification and combination of the BPS health domains. Existing risk assessment tools for older people, for example the Risk Instrument for Screening in the Community (RISC) [57, 58] focus heavily on prioritizing the bio-functional domain. Many such studies can be classed as similar to well-known measures such as the EQ-5D [59] and SF-36 [60] which can be argued seek to capture Health-Related Quality of Life (H-R QoL) [61] in adults. For example, by directly capturing the extent to which bio-medical conditions may interfere with social life, rather than social life itself being conceived as a health domain.

Other tools, such as EASYCare and the WHOQOL-BREF [62], aim to be more inclusive and balanced in their conception of all three BPS domains, and can be used to appraise needs as well as combined into latent and more holistic outcomes. Still other types of measures, termed simply ‘Quality of life’ (QoL) or well-being outcomes can be defined as theoretically distinct from bio-functional health, and thus will prioritize the psychosocial. CASP-19 defined as composed of control, autonomy, self-realization and pleasure domains [63] and the ageing life satisfaction index-wellbeing [64] are examples of these. Such tools can be usefully used to examine effects of bio-functional limitations on psychosocial outcomes.

In contrast to existing measures, the BPS Risk Screener is uniquely dynamic, and gives equal consideration to all the BPS domains of health. It aims to reach older people *before* the onset of critical and limiting diseases as much as during, and does not assume that those without illness are not vulnerable. The tool can be used in combination with EASYCare’s original tool and design, which includes a section asking to agree health needs and priorities; completing this section with the participant will provide more detailed qualitative support to combine with the Screener’s scores.

The Screener’s main purpose is not to replace human judgement, empathy or clinical experience. When properly administered, it is conceived as a tool to help guide decisions on finding the right program and services to match the needs of the older person, using the BPS combined managing score as a guide. The individual B, P, S managing counts can be used to help to further inform care planning, mapping areas of need to more in-depth assessment or referrals.

For example, in the ComSA program, a more vulnerable adult would be linked to Care Management and depending on their individual domain needs may be referred to either or all: community-based nursing and primary care (B), counselling and support services (P), and community inclusion activities (S). More proactive Third Agers would be recommended to the ComSA Community Development activities, including: self-care and health maintenance classes (B), guided autobiography therapeutic groups (P), and social and interest groups (S). In devising the tool, the BPS managing score was also intended to provide validated, robust and change sensitive outcomes with which to evaluate interventions.

### Summary of findings

The study shows that overall, the risk-screening tool and proposed novel scoring system is clearly able to identify older people who are at increased risk of poor managing outcomes due to additive effects of BPS health loads. The most vulnerable older people were indeed consistently identified at 4 pts. and upwards of the managing score, although we recommend this threshold be used with caution; providing a shared concept as well as language to inform care of people according to where their needs will rank on a spectrum. Not as an absolute criterion for program inclusion.

This work builds on what we know about social health, often referred to simply as isolation and poor relationships. We know these play important roles in the risk of mortality [48] and morbidity [24, 65]. Yet, traditionally little consideration has been given to explicitly defining, and embedding this construct into health models as a distinct domain in itself. Our study demonstrates that, similar to the P domain, the S component of health may appear small as a stand-alone part, but when treated as an additive in a load, it does significantly contribute to health problems adding up.

BPS Risk screener’s greatest attribute is that it renders programming transparent. The current set of analyses tells us, for instance, that if the goal is only to reduce LoS and ED visits the S and B domains are important. On the other hand, if the goal is to design programs to reduce hospitalization itself, then let us focus on lifting the B. If falls are the outcome programmer most want to address, then let is lift the B and P, and so on. These associations are however likely to change across cultural contexts, and remain to be tested in different settings. Moreover, we must remember that improving the general experience of health at older ages, and helping older people to simply manage better, has been clearly demonstrated to require a fully BPS and integrated approach.

### Strengths, limitations and future work

This study is underpinned by a contemporary paradigm, transferred across developmental studies of resilience, which assumes older peoples continued capability for growth. It operationalizes BPS health as a dynamic process and treats vulnerability as modifiable and successful ageing as achievable, even in the Fourth Age. Although our B, P and S clusters were confirmed, and our BPS health load concept and scoring approach supported, we noted that existing items could still be better adapted to local settings, and expanded. For instance, more explicit and culturally better-worded questions might clarify the B domain. It was notable for instance that neither sleep problems nor pain were retained in this cluster, as these are big B issues at older ages. This may be due to unclear wording and/or interpretations that overlap between the B (pain) and P (anxiety) domains.

The P domain was also limited in items and may not capture more acute emotional experiences well. As for the S domain, Lubben prioritizes family relationships outside the home. However, in Singapore most families are close knit and often live together. As such, we realized during our risk screener administration that Lubben did not capture the impact of prominent and important familial relationships in the home. Also, EASYCare’s structure, could be refined to incorporate meaningful S items and better capture P wellness.

Our outcomes did not account for intermediate to long-term care, neither for nursing home facilitation or hospitalization for mental health care. Moreover, we were lacking QoL (e.g. CASP-19) as an outcome, which should be added to further testing of this tool. A major limitation of this study is the natural self-selection phenomena that the very vulnerable will be hard to find, let alone agree to be interviewed. The low numbers in the highest risk bands resulted in wide confidence intervals and could have contributed to some instability of analysis at that level.

This study followed a cross-sectional design, limited to one neighbourhood in Singapore, although the study population profile was generally well matched in comparison to national census data. Further study is required for confirmatory factor analysis, and to examine the reliability of the tool, and using it as a measure of change and programmatic evaluation outcome; the current study simply explores the BPS domains and tests the utility of the managing score categories.

## Conclusions

In closing, this work answers the recent call for innovation in measurement and management toward integrity of health systems [66]. It pays particular attention to the fact that community health programs differ from other health innovations in that they naturally do not just focus on a single condition, setting, service provider or process, but instead seek to optimize the overall health and functional status of clients, while curtailing excessive or avoidable healthcare use [67]. Such systems are predisposed to, and in need of evidenced based approaches to maximise integration. A focus on understanding BPS dynamics and the mix of managing or risk profiles, creates a common understanding and dialogue around risk, and is an important starting point for geriatric assessment, and the basis for engendering Adaptive Capability.

Moreover, given that psychosocial risks may be more malleable than bio-functional ones, especially to older old people in the fourth age, we anticipate that tools that accurately capture P and S risks will be more change sensitive, and such measures should be encouraged and improved. In order to improve the BPS Risk Screener and address the gaps in questionnaire items identified in the current work, a complementary mixed-method study, refining and testing new BPS items into an updated, more cohesive scale is forthcoming.

## Additional files

Additional file 1: Figure S1. ‘How can we help?’ linking patients to suitable interventions, using biopsychosocial risk screening toward enabling successfully ageing in place. Conceptual mapping (no data). (DOCX 86 kb)

Additional file 2: Figure S2. Describing Loads-Levers-Lifts process model toward Adaptive Capability. Conceptual mapping (no data). (PPTX 234 kb)

Additional file 3: Figure S3a and b. Scoring of biopsychosocial health to capture additive effects or health loads within and across BPS domains. Descriptive process (no data). (DOCX 14 kb)

Additional file 4: Table S1. Whampoa survey respondents compared to Singapore resident population ^a^, for over 60 years, in absolute numbers and (%).Compared the Whampoa data collected during August to October 2014, using non-randomized convenience sampling (*n* = 1325, 60 years of age and older) to Singapore census data (60 years of age and older). Data derived from: Census data, The Yearbook of Statistics Singapore, 2015; and Population trends, 2015. Department of Statistics Singapore. (DOCX 76 kb)

Additional file 5: Figure S4. Distribution of managing scores, *n* = 1325 study participants over 60 years of age. Data was collected during August to October 2014, using non-randomized convenience sampling, and listings of addresses of participants of 60 years of age and older. (DOCX 57 kb)

Additional file 6: Figure S5a-d. Managing scores by outcomes of interest, n = 1325 study participants over 60 years of age. Data was collected during August to October 2014, using non-randomized convenience sampling, and listings of addresses of participants of 60 years of age and older. (DOCX 2557 kb)

Additional file 7: Figure S5e-h. Managing scores by outcomes of interest, n = 1325 study participants over 60 years of age. Data was collected during August to October 2014, using non-randomized convenience sampling, and listings of addresses of participants of 60 years of age and older. (DOCX 163 kb)

Additional file 8: Figure S6. Summary of multivariate associations by outcomes of interest. Summary of results based on study participants. Data was collected during August to October 2014, using non-randomized convenience sampling, and listings of addresses of participants of 60 years of age and older. (DOCX 2475 kb)

## Abbreviations

BPS: BioPyschoSocial
CASP: Control, Autonomy, Self-realization, Pleasure
ComSA: Community for successful ageing
ED: Emergency department
H-R QoL: Health-related quality of life
LoS: Length of stay
Qol: Quality of life
WHO: World Health Organization
