# Supplementary figures and images for: The theoretical and empirical basis of a BioPsychoSocial (BPS) risk screener for detection of older people’s health related needs, planning of community programs, and targeted care interventions
Source: BMC Geriatr. 2018 Feb 17;18:49. doi: 10.1186/s12877-018-0739-x (PMC5816546; doi:10.1186/s12877-018-0739-x)

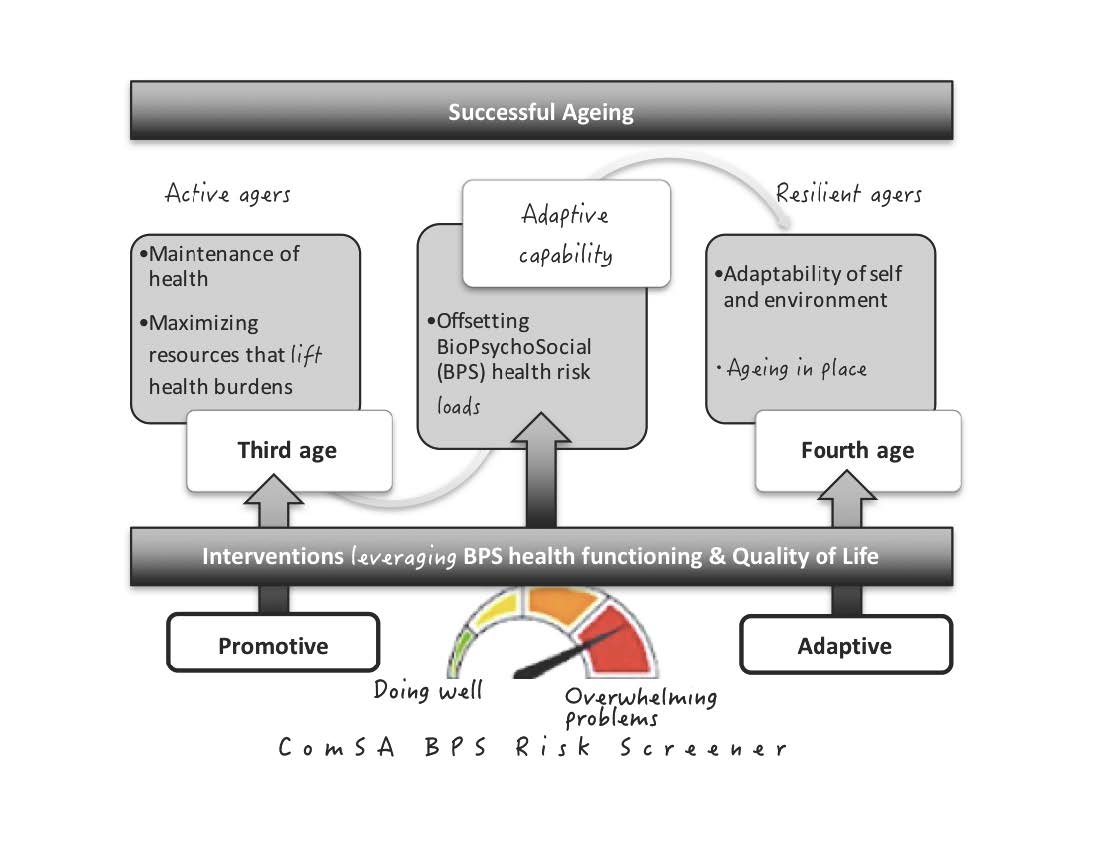

Supplement: Supplementary file 1 — ‘How can we help?’ linking patients to suitable interventions, using biopsychosocial risk screening toward enabling successfully ageing in place. Conceptual mapping (no data). (DOCX 86 kb) [file 12877_2018_739_MOESM1_ESM.docx]

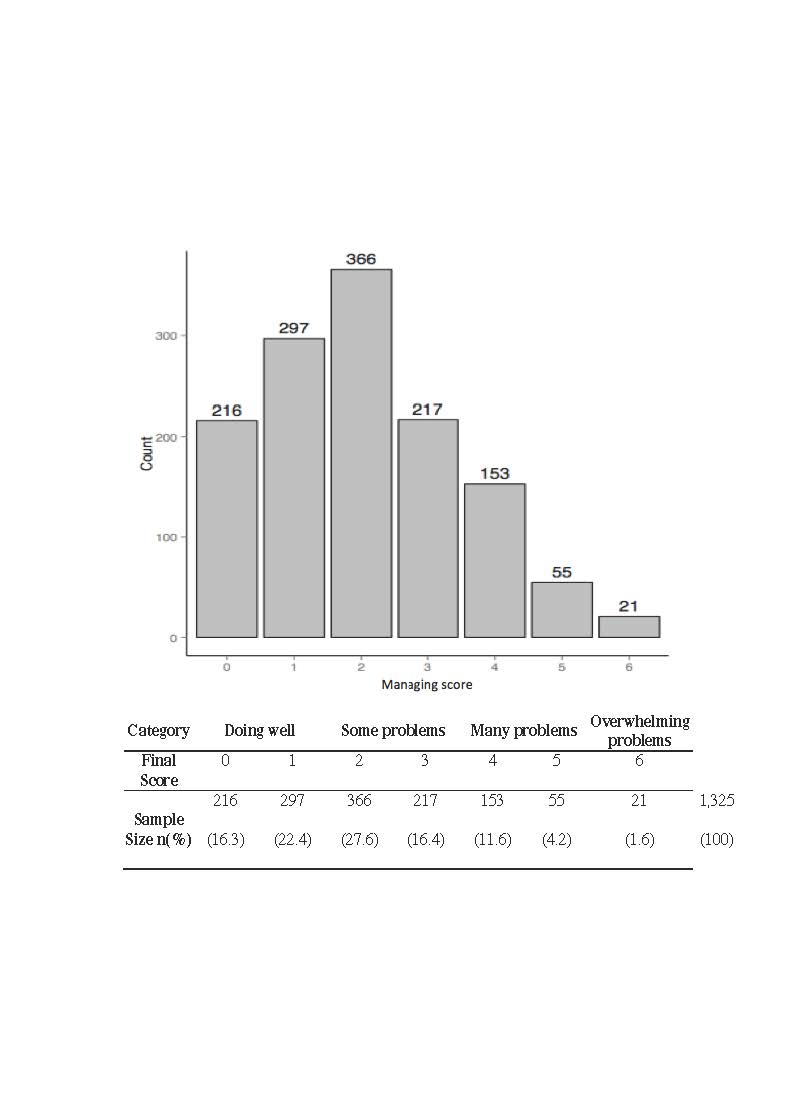

Supplement: Supplementary file 5 — Distribution of managing scores, n = 1325 study participants over 60 years of age. Data was collected during August to October 2014, using non-randomized convenience sampling, and listings of addresses of participants of 60 years of age and older. (DOCX 57 kb) [file 12877_2018_739_MOESM5_ESM.docx]

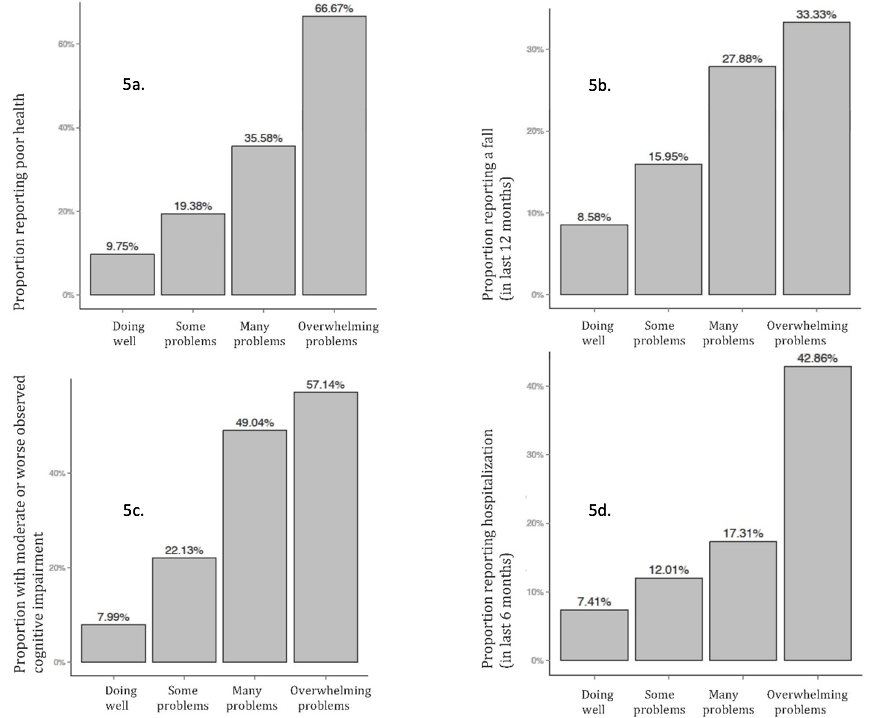

Supplement: Supplementary file 6 — Managing scores by outcomes of interest, n = 1325 study participants over 60 years of age. Data was collected during August to October 2014, using non-randomized convenience sampling, and listings of addresses of participants of 60 years of age and older. (DOCX 2557 kb) [file 12877_2018_739_MOESM6_ESM.docx]

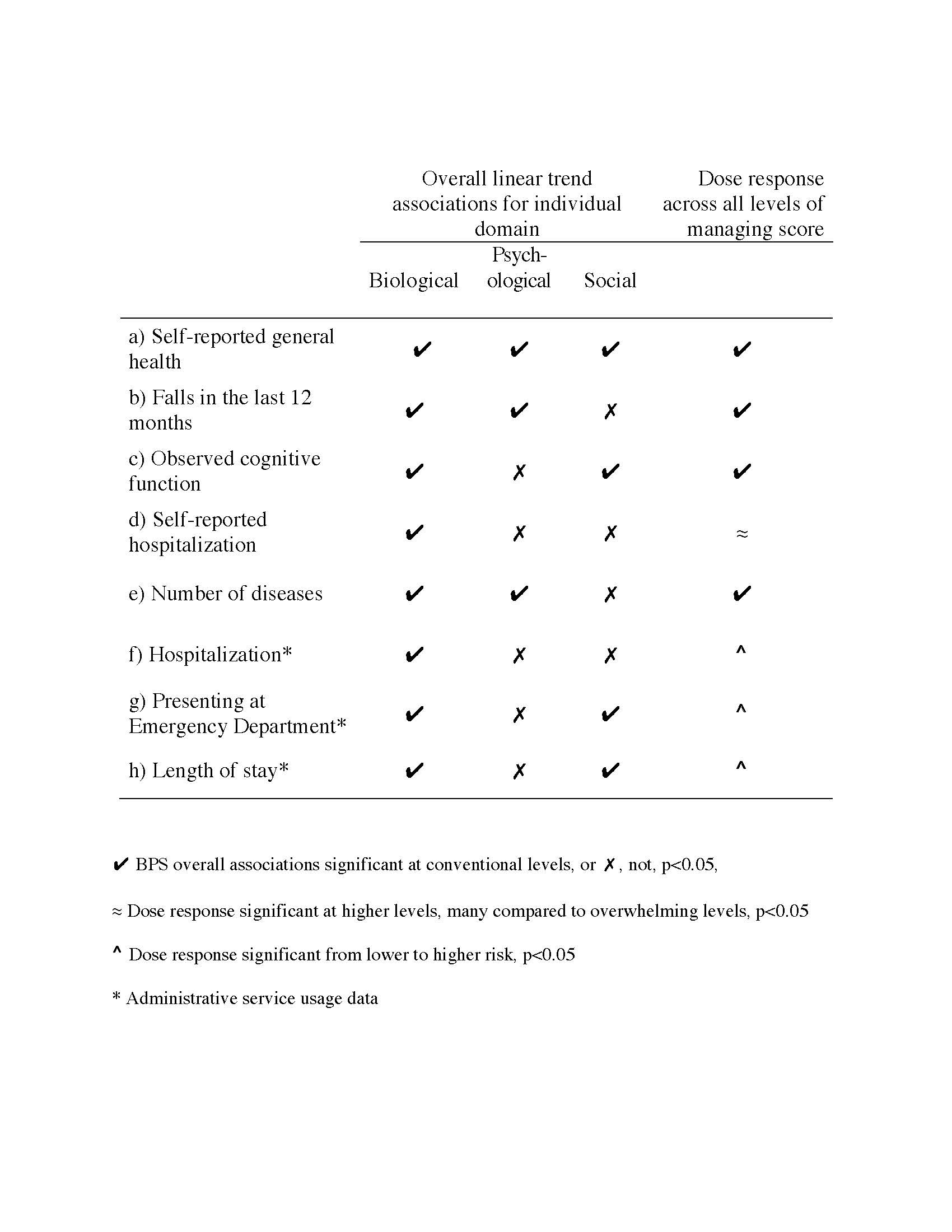

Supplement: Supplementary file 7 — Managing scores by outcomes of interest, n = 1325 study participants over 60 years of age. Data was collected during August to October 2014, using non-randomized convenience sampling, and listings of addresses of participants of 60 years of age and older. (DOCX 163 kb) [file 12877_2018_739_MOESM7_ESM.docx]

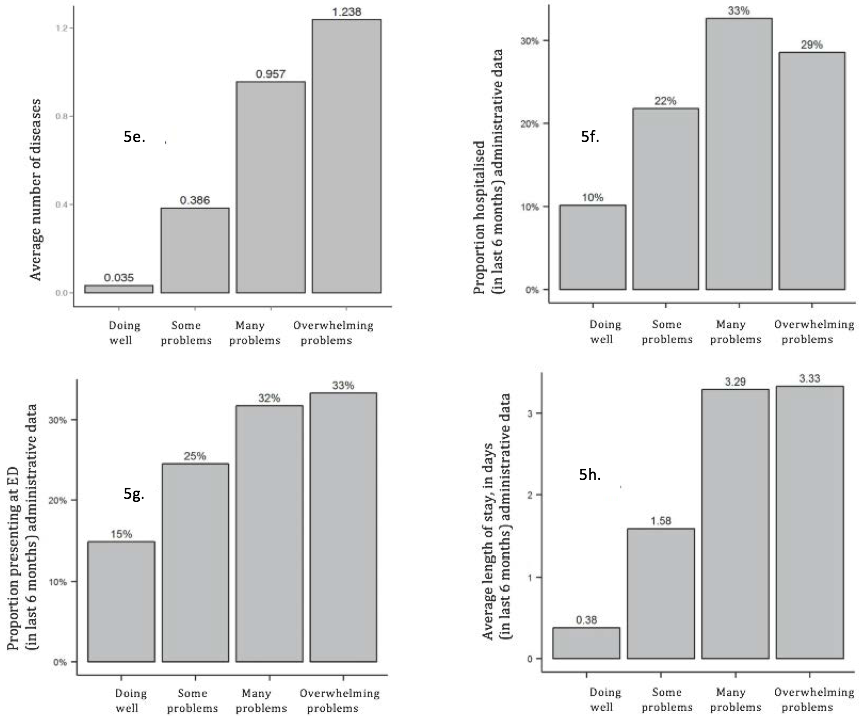

Supplement: Supplementary file 8 — Summary of multivariate associations by outcomes of interest. Summary of results based on study participants. Data was collected during August to October 2014, using non-randomized convenience sampling, and listings of addresses of participants of 60 years of age and older. (DOCX 2475 kb) [file 12877_2018_739_MOESM8_ESM.docx]
