# Supplementary material for: The theoretical and empirical basis of a BioPsychoSocial (BPS) risk screener for detection of older people’s health related needs, planning of community programs, and targeted care interventions
Source: BMC Geriatr. 2018 Feb 17;18:49. doi: 10.1186/s12877-018-0739-x (PMC5816546; doi:10.1186/s12877-018-0739-x)
Supplement: Supplementary file 2 — Describing Loads-Levers-Lifts process model toward Adaptive Capability. Conceptual mapping (no data). (PPTX 234 kb) [file 12877_2018_739_MOESM2_ESM.pptx]

## Slide 1
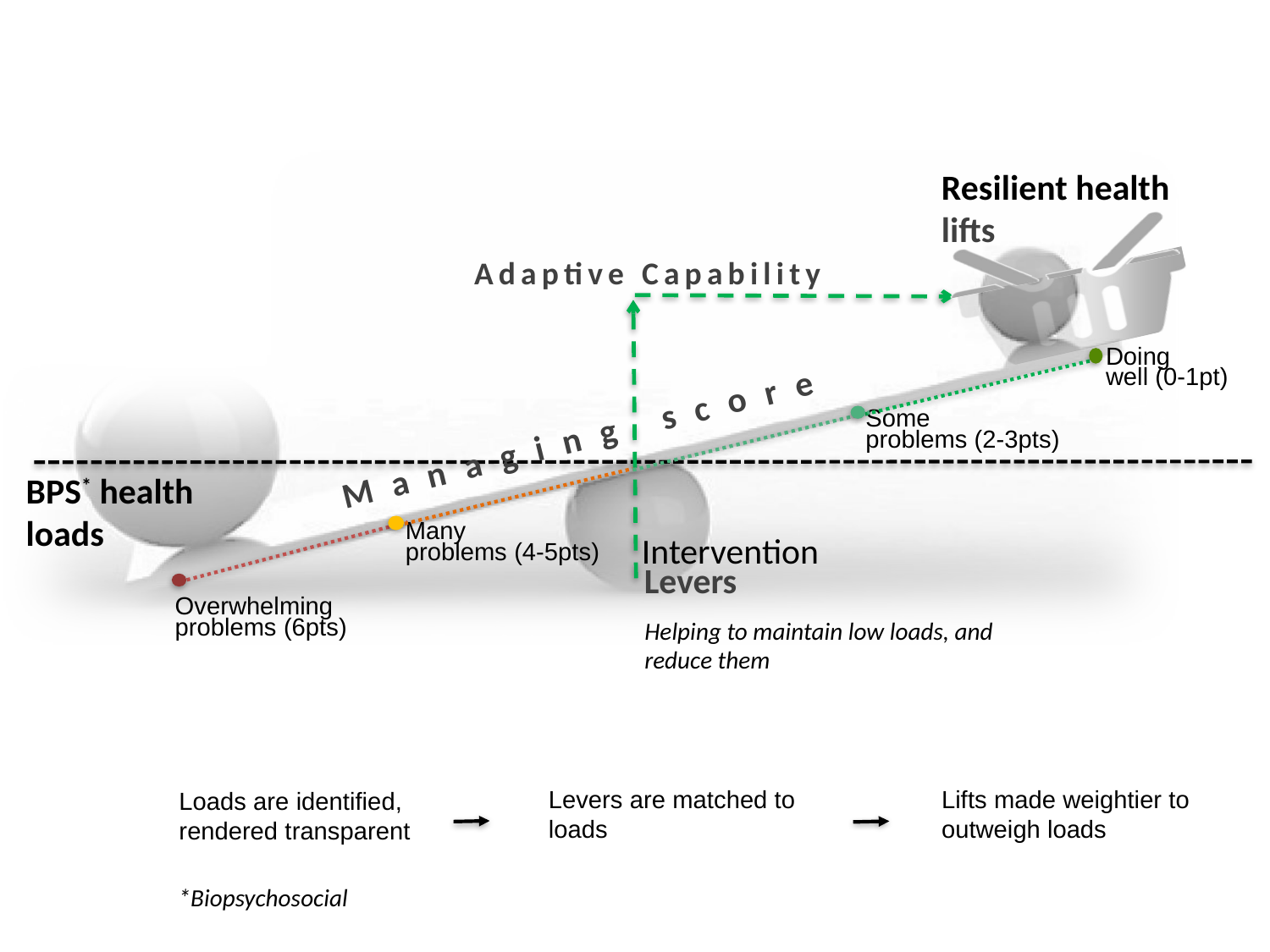

Resilient health lifts
Adaptive Capability
Doing
well (0-1pt)
Managing score
Some
problems (2-3pts)
BPS* health
loads
Many
problems (4-5pts)
Intervention
Levers
Overwhelming problems (6pts)
Helping to maintain low loads, and reduce them
Lifts made weightier to outweigh loads
Levers are matched to loads
Loads are identified, rendered transparent
*Biopsychosocial
