# Supplementary material for: The theoretical and empirical basis of a BioPsychoSocial (BPS) risk screener for detection of older people’s health related needs, planning of community programs, and targeted care interventions
Source: BMC Geriatr. 2018 Feb 17;18:49. doi: 10.1186/s12877-018-0739-x (PMC5816546; doi:10.1186/s12877-018-0739-x)
Supplement: Supplementary file 3 — and b. Scoring of biopsychosocial health to capture additive effects or health loads within and across BPS domains. Descriptive process (no data). (DOCX 14 kb) [file 12877_2018_739_MOESM3_ESM.docx]

| BioPsychoSocial domains | Problems | Domain Managing  counts |
| --- | --- | --- |
| Biological Health | None | 0 |
|  | Some | 1 |
|  | A lot | 2 |
| Psychological Health | None | 0 |
|  | Some | 1 |
|  | A lot | 2 |
| Social health | None | 0 |
|  | Some | 1 |
|  | A lot | 2 |

3a

| Managing Score | Derived from domain specific  Managing counts |
| --- | --- |
| 0-1 means: | Doing well |
| 2-3 means: | Some problems |
| 4-5 means: | Many problems |
| 6 equates to: | Overwhelming problems |

3b
