# Supplementary material for: The theoretical and empirical basis of a BioPsychoSocial (BPS) risk screener for detection of older people’s health related needs, planning of community programs, and targeted care interventions
Source: BMC Geriatr. 2018 Feb 17;18:49. doi: 10.1186/s12877-018-0739-x (PMC5816546; doi:10.1186/s12877-018-0739-x)
Supplement: Supplementary file 4 — Whampoa survey respondents compared to Singapore resident population a, for over 60 years, in absolute numbers and (%).Compared the Whampoa data collected during August to October 2014, using non-randomized convenience sampling (n = 1325, 60 years of age and older) to Singapore census data (60 years of age and older). Data derived from: Census data, The Yearbook of Statistics Singapore, 2015; and Population trends, 2015. Department of Statistics Singapore. (DOCX 76 kb) [file 12877_2018_739_MOESM4_ESM.docx]

Supplementary file Table 1: Whampoa survey respondents compared to Singapore resident population ^a^, for over 60 years, in absolute numbers and (%)

| Age ^b^ | 60 - 64 | 65 - 69 | | | 70 - 74 | 75 – 79 | 80 - 84 | | 85+ | Total | |
| --- | --- | --- | --- | --- | --- | --- | --- | --- | --- | --- | --- |
| Singapore in ‘000 | 231.5  (34.91) | 161.2  (24.31) | | | 106.8  (16.11) | 76.3  (11.51) | 48.4  (7.30) | | 38.9  (5.86) | 663.1  (100) | |
| Whampoa | 258  (19.47) | 322  (24.30) | | | 273  (20.60) | 230  (17.36) | 123  (9.28) | | 119  (8.98) | 1325  (100) | |
| Ethnic Group ^b^ | Chinese | | | Malay | | Indian | | Other | | Total | |
| Singapore in ‘000 | 550.8  (83.06) | | | 62.6  (9.45) | | 400.8  (6.04) | | 9.6  (1.45) | | 663.1  (100) | |
| Whampoa | 1106  (83.47) | | | 55  (4.15) | | 145  (10.94) | | 19  (1.43) | | 1325  (100) | |
| Gender ^b^ | Males | | Females | | | | | | | Total | |
| Singapore in ‘000 | 309.1  (46.62) | | 354.0  (53.38) | | | | | | | 663.1  (100) | |
| Whampoa | 545  (41.13) | | 780  (58.87) | | | | | | | 1325  (100) | |
| Education ^c^ | Under  Secondary | | Secondary | | | Post-Secondary | | | | Total | |
| Singapore in ‘000 | 457.5  (63.74) | | 131.7  (18.35) | | | 128.6  (17.92) | | | | 717.7  (100) | |
| Whampoa | 820  (61.89) | | 383  (28.91) | | | 122  (9.21) | | | | 1325  (100) | |
|  | | | | | | | | | | |  |

1. The Yearbook of Statistics Singapore, 2015: <http://www.singstat.gov.sg/docs/default-source/default-document-library/publications/publications_and_papers/reference/yearbook_2015/yos2015.pdf>
2. Population Trends 2015, Singapore Statistics, table A1.5, <http://www.singstat.gov.sg/docs/default-source/default-document-library/publications/publications_and_papers/population_and_population_structure/population2015.pdf>
3. Singapore resident population comprises Singapore citizens and permanent residents
